# Inferior pancreaticoduodenal artery aneurysm: endovascular approach

**DOI:** 10.1590/1677-5449.200101

**Published:** 2021-05-10

**Authors:** Patrick Bastos Metzger, Kamilla Rosales Costa, Simone Lessa e Silva, Alan Costa Gouveia, Murilo Quadro Berbert, Milton Oliveira de Albuquerque Mello, Fabrício Mascarenhas de Oliveira, Ricardo Fernandes Ferraz Melo

**Affiliations:** 1 Escola Bahiana de Medicina e Saúde Pública – EBMSP, Salvador, BA, Brasil.; 2 Obra Sociais Irmã Dulce – OSID, Hospital Santo Antônio, Salvador, BA, Brasil.; 3 Hospital Português, Salvador, BA, Brasil.; 4 Hospital da Bahia – HBA, Salvador, BA, Brasil.

**Keywords:** aneurysm, endovascular procedures, therapeutic embolization

## Abstract

Aneurysms of the pancreaticoduodenal arteries are a rare condition. In the majority of cases, diagnosis is made in emergency situations due to complications such as rupture, which is associated with high mortality rates (21-26%). Embolization of the aneurysm sac is the treatment of choice, because of its high efficacy and lower mortality. This article presents and discusses a case of inferior pancreaticoduodenal artery aneurysm that was diagnosed during investigation of gastrointestinal symptoms. The treatment provided was microcoil embolization, with complete exclusion of the aneurysm and a good clinical course.

## INTRODUCTION

Aneurysms of the visceral arteries (VAA) are rare, with an incidence of 0.02% in the general population.^1^^-^ 3 Aneurysms of the pancreaticoduodenal artery (PDAA) are the least frequent type, accounting for just 2% of all VAA,^4^ and inferior pancreaticoduodenal artery aneurysms (iPDAA) are the least often described subtype.^1^^-^ 3

Use of angiotomography has increased the number of PDAA diagnoses, as a result of incidental findings.^1^^-^ 3 Nonetheless, the majority of PDAA are still discovered and treated in emergency situations, with increased risk of complications such as hemoperitoneum and hypovolemic shock, with rates of aneurysm rupture in the literature in the range of 45-53%.^3^^-^ 5 These aneurysms are supplied by collateral vessels with hyperdynamic flow and are often associated with occlusive stenosis of the celiac trunk (CT) and superior mesenteric artery (SMA).^3^^,^ 4

Endovascular treatment is the preferred method, except in cases of suspected visceral ischemia, in patients with hostile vascular anatomy, or if prior endovascular treatment has failed. In such cases, an open approach is indicated.^2^^,^ 3

We report on endovascular treatment of a large true IPDAA without significant stenosis (stenosis > 60%) of the main arterial trunks, using superselective navigation with microcatheters and embolization with microcoils.

## CASE DESCRIPTION

**Figure 1:**
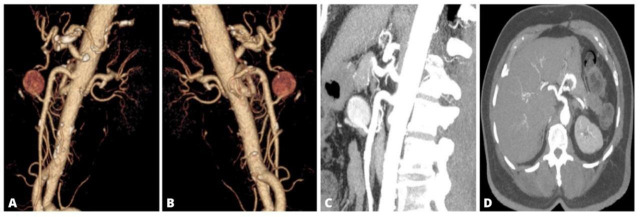
(A) Left anterior oblique volumetric angiotomographic reconstruction showing a large inferior pancreaticoduodenal artery aneurysm; (B) Right anterior oblique volumetric angiotomographic reconstruction showing communications with the celiac trunk (CT) via the gastroduodenal artery and with the superior mesenteric artery via the inferior pancreaticoduodenal artery; (C) Axial angiotomographic slice showing the ostial plaque in the CT; (D) Sagittal angiotomographic slice showing the ostial plaque in the CT causing approximately 20% stenosis.

A 62-year-old, hypertensive woman, with type 2 diabetes, obesity, and dyslipidemia, was undergoing ambulatory investigation for postprandial fullness with a gastroenterologist. She had no family or personal history of rheumatic diseases, episodes of digestive hemorrhage, postprandial pain, abdominal traumas, alcoholic beverage consumption, pancreatitis, or recent weight loss. Angiotomography showed a large IPDAA, with dimensions of 54 x 60 mm, connecting the SMA to the gastroduodenal artery. The SMA did not have ostial plaque, while the CT had ostial plaque causing stenosis estimated at approximately 20% (Figure 1). A transthoracic echocardiogram found no evidence of pathological changes.

**Figure 2:**
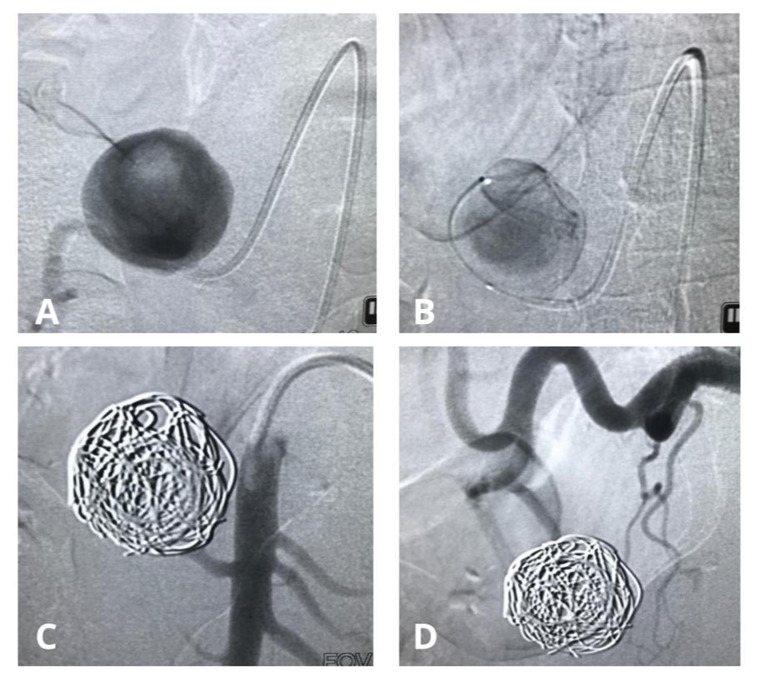
(A) Arteriography via the superior mesenteric artery (SMA), with selective catheterization; (B) Microcatheter selection of the large aneurysm of the inferior pancreaticoduodenal artery; (C) Selective arteriography of the SMA after embolization with microcoils, showing complete exclusion of the aneurysm sac; (D) Selective arteriography of the celiac trunk after embolization with microcoils, showing complete exclusion of the aneurysm sac.

The management option chosen was arteriography followed by superselective navigation with microcatheters and embolization with microcoils. The procedure was performed in the angiography suite, under local anesthesia and conscious sedation, with femoral access and a 5 Fr introducer (Terumo Medical, Somerset, United States). After arteriographic study with sequential and selective catheterization of the CT and SMA, the decision was taken to catheterize the SMA and perform superselective navigation with a microcatheter via the inferior pancreaticoduodenal artery to the large aneurysm. Embolization was performed using a Rebar-18® microcatheter (ev3; Plymouth, United States) and a SilverSpeed-16® microguidewire (ev3; Plymouth, Minnesota, United States), followed by embolization with 6 18 mm x 40 cm Axium® Prime 3D detachable microcoils (ev3; Plymouth, United States). After complete embolization of the aneurysm, a second selective arteriographic study via the CT and SMA showed complete exclusion of the IPDAA and improved perfusion of the intestinal loops (Figure 2).

**Figure 3:**
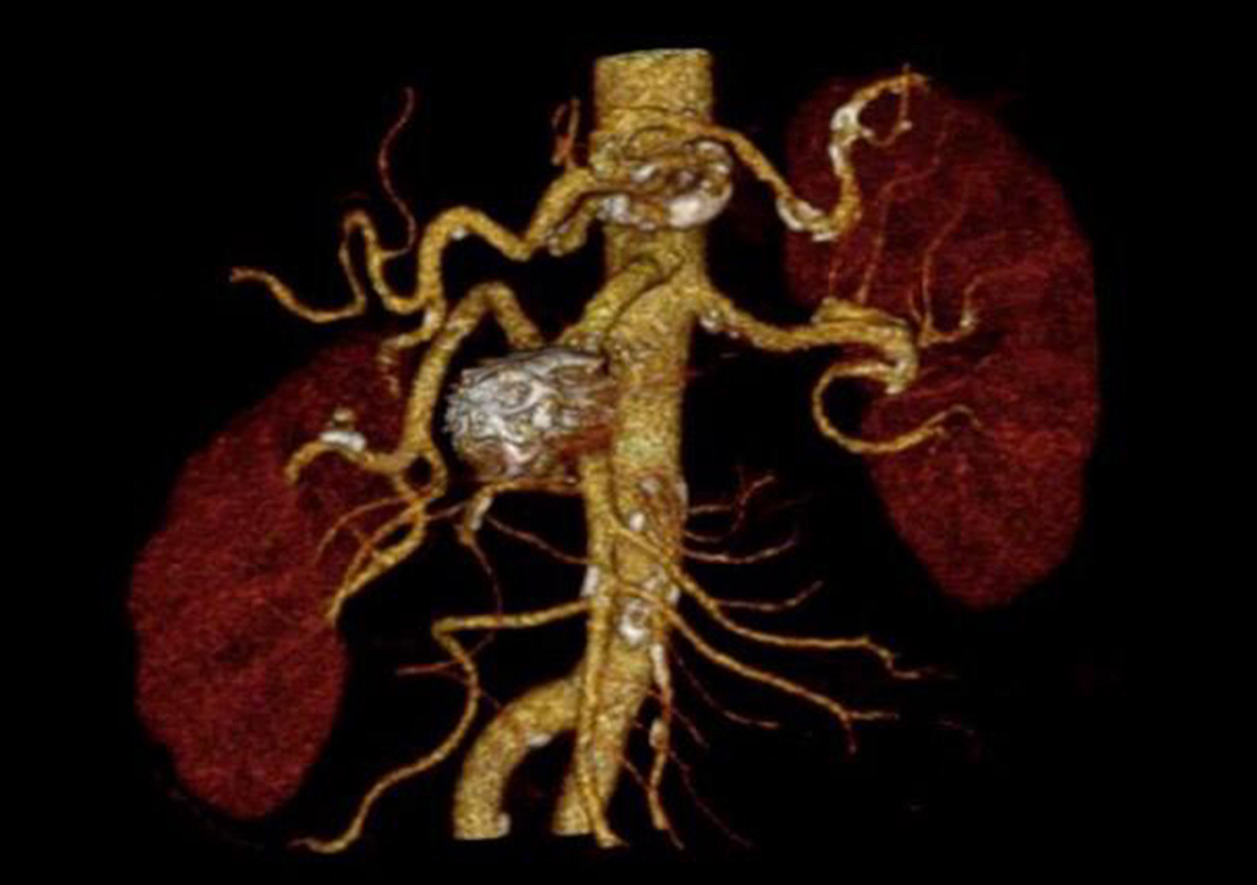
Postoperative volumetric angiotomographic reconstruction showing no filling of the aneurysm, which has been completely excluded.

Postoperative monitoring took place in the intensive care unit, with good progress and hospital discharge on the second day after the procedure. Control angiotomography at 1 month showed the SMA and CT were patent, with complete exclusion of the IPDAA. At 1-year outpatients follow-up, the patient reported resolution of the postprandial complaints described previously (Figure 3).

## DISCUSSION

Inferior pancreaticoduodenal artery aneurysms are extremely rare, but are probably undernotified, since symptoms are nonspecific and the location makes diagnosis less likely.^5^^,^ 6 They affect both sexes with equal incidence,^5^^,^ 6 and mean age of greatest diagnostic frequency is 60 years.^6^ The primary cause of true IPDAAs is occlusion or stenosis of one of the main arterial trunks, whether the SMA or the CT, the second of which is more often involved (80%); these aneurysms are related to occlusive arterial disease involving the ostia of the trunk arteries in 50-80% of cases.^3^^,^ 7 ^,^ 8 After this event, the velocity of blood flow to the pancreatic arterial arches increases, causing progressive dilation of the arterial lumen (the hyperdynamic collateral circulation hypothesis).^2^^,^ 5 ^,^ 8 ^,^ 9 Other reported causes are atherosclerosis, connective tissue diseases, and infections.^5^^,^ 10 For many years the last of these were considered the primary cause of IPDAA (60%), but recent studies have shown that atherosclerotic disease is the most common cause and infectious etiology is responsible for 4.8% of cases diagnosed, rather than over half, as was previously thought.^10^ The patient in this case was diagnosed with a true IPDAA, since she did not meet the criteria for suspected mycotic infection or connective tissue diseases and also did not have ostial stenosis of the CT or SMA, ruling out the hyperdynamic collateral circulation hypothesis.^2^^,^ 5 ^,^ 8 ^,^ 9 In view of this, the probable cause of this patient’s aneurysm is atherosclerotic disease.

The principal complication associated with IPDAA is rupture, with incidence and mortality rates in the range of 45-62% and 21-26%, respectively.^3^^,^ 11 ^,^ 12 A significant proportion of diagnoses of this condition are made in emergencies, with symptoms of epigastralgia or abdominal pains that radiate to the back and may be accompanied by signs of shock or hemoperitoneum secondary to aneurysmal rupture.^10^ This complication is independent of aneurysm size.^9^

The patient described had a gastrointestinal complaint of postprandial fullness under ambulatory investigation, leading to the diagnosis of IPDAA after abdominal angiotomography. Nonspecific abdominal pains, nausea, vomiting, jaundice, and gastrointestinal bleeding are symptoms that may be present in these cases. Aneurysms of infectious origin may cause fever. On physical examination, a pulsating abdominal mass or abdominal murmur may be found, but are rare. The frequent presence of these manifestations in patients with IPDAA distinguishes them from those with other types of aneurysms of splanchnic arteries, the majority of which are asymptomatic (90%);^10^ however, in the majority of cases, diagnosis is made after rupture of the aneurysm. In view of their elevated mortality, VAA should be part of the differential diagnosis for all patients with sudden abdominal pains combined with anemia.^9^ Work-up examinations that are useful for diagnosis include abdominal Doppler ultrasonography, which is an inexpensive and accessible method; magnetic resonance angiography; and angiotomography (the gold standard), which provides the information necessary for both diagnosis and treatment planning.^9^^,^ 10

Since the condition is rare, treatment indications are controversial in the literature and it is necessary to evaluate factors such as age, clinical presentation, aneurysm characteristics, associated comorbidities, and risk factors.^3^^,^ 5 ^,^ 10 There is some consensus on the indications for repair of true aneurysms: diameter exceeding 20-25 mm, even if asymptomatic; symptomatic patients; female patients of reproductive age; and aneurysms associated with inflammatory or infectious processes, connective tissue disease, vasculitis, or congenital diseases, because of the high risk of rupture.^5^^,^ 6 ^,^ 9 ^,^ 13 ^,^ 14 Pseudoaneurysms should be treated immediately after they are identified, because of the elevated risk of rupture.^5^^,^ 7 ^,^ 9 Available treatment options include conventional surgical treatment (open repair) and endovascular approaches. Conventional surgery is indicated in hemodynamically unstable patients, when there is a need for revascularization of blood flow, and in cases of aneurysms in territories that are inaccessible to endovascular technique or after failure of embolization. The technique consists of proximal and distal ligature of the artery, exeresis of the aneurysm, and reconstruction of the artery. In addition to being a more invasive procedure, open repair involves limitations caused by difficulties with accessing the aneurysm and is associated with mortality rates in the range of 15-23.7%.^5^^,^ 7 ^,^ 10

As the techniques and devices available have evolved, endovascular treatment with embolization of the aneurysm has become the treatment of choice in these cases, due to its effectiveness (67-100%), lower mortality rate (2.7%), lower risk of rupture, less postoperative pain, and shorter recovery time after the procedure, with consequent earlier return to daily activities, and it is also an option for patients with a history of abdominal surgery who have a hostile abdomen.^5^^,^ 9 ^,^ 10 However, certain determinants limit its use: low availability in emergency centers, risk of injury secondary to access, and embolization of target organs, in addition to the high cost.^9^

Embolization options include use of cyanoacrylate, coils, or covered or conventional stents, which can be combined in some situations.^7^^,^ 9 ^,^ 10 Other percutaneous treatment options are flow diversion and percutaneous thrombin injection.^9^^,^ 15 ^,^ 16 The choice of technique is dependent on the need to preserve the feeder artery, on anatomic characteristics, and on the type of aneurysm, in addition to the degree of tortuosity of the native artery. Adequate treatment of the aneurysm is guaranteed by complete embolization of all entry and exit arteries.^9^^,^ 15 ^,^ 16

The patient in this case had an oligosymptomatic IPDAA with dimensions of 54 x 60 mm that had not ruptured. Since she was compatible with the criteria for indication of the treatment approach most supported in the literature, and also because of the high risk of rupture, elective treatment using endovascular techniques was indicated, performed by embolization with 6 Axium® Prime 3D detachable microcoils (ev3; Plymouth, Minnesota, USA), achieving permanent thrombosis of the aneurysm sac and its exclusion. Embolization was total, as confirmed intraoperatively by selective angiography of the CT and SMA, with improved intestinal blood flow and good postoperative recovery, including resolution of the preexisting clinical symptoms. Control angiotomography at 1 month confirmed total thrombosis of the aneurysm and found no evidence of filling.
