# Supplementary material for: Inferior pancreaticoduodenal artery aneurysm: endovascular approach
Source: J Vasc Bras. 2021 May 10;20:e20200101. doi: 10.1590/1677-5449.200101 (PMC8147707; doi:10.1590/1677-5449.200101)
Supplement: Translation

# Translation: Aneurisma de artéria pancreatoduodenal inferior: tratamento endovascular

**Authors:** Patrick Bastos Metzger, Kamilla Rosales Costa, Simone Lessa e Silva, Alan Costa Gouveia, Murilo Quadro Berbert, Milton Oliveira de Albuquerque Mello, Fabrício Mascarenhas de Oliveira, Ricardo Fernandes Ferraz Melo

**Affiliations:** 1 Escola Bahiana de Medicina e Saúde Pública – EBMSP, Salvador, BA, Brasil.; 2 Obra Sociais Irmã Dulce – OSID, Hospital Santo Antônio, Salvador, BA, Brasil.; 3 Hospital Português, Salvador, BA, Brasil.; 4 Hospital da Bahia – HBA, Salvador, BA, Brasil.

**Keywords:** aneurisma, procedimentos endovasculares, embolização terapêutica

## Abstract

Os aneurismas das artérias pancreatoduodenais constituem uma afecção rara. Seu diagnóstico é realizado, na maioria das vezes, em situações de emergência por complicações como a rotura, a qual está associada a altas taxas de mortalidade (21-26%). A embolização do saco aneurismático é o tratamento de escolha, devido à sua alta efetividade e menor mortalidade. Neste artigo, é apresentado e discutido um caso de aneurisma de artéria pancreatoduodenal inferior com diagnóstico obtido durante investigação de sintomas gastrointestinais. O tratamento instituído foi a embolização com micromolas, com completa exclusão do aneurisma e boa evolução clínica.

## INTRODUÇÃO

Os aneurismas de artérias viscerais (AAV) são raros, com uma incidência de 0,02% na população geral^1^^-^ 3. Entre eles, os aneurismas da artéria pancreatoduodenal (AAPD) são os menos frequentes, correspondendo a apenas 2% de todos os AAV^4^, sendo os aneurismas da artéria pancreatoduodenal inferior (AAPDi) os menos relatados^1^^-^ 3.

O uso da angiotomografia tem aumentado o diagnóstico de AAPD, devido a descobertas incidentais^1^^-^ 3. Por outro lado, atualmente a maioria dos AAPD ainda são descobertos e tratados em situações de emergência, com maior risco de complicações como hemoperitôneo e choque hipovolêmico, tendo taxas de ruptura aneurismática entre 45-53%, na literatura^3^^-^ 5. Esses aneurismas são supridos por ramos colaterais com fluxo hiperdinâmico e estão frequentemente associados a estenoses oclusivas do tronco celíaco (TC) e da artéria mesentérica superior (AMS)^3^^,^ 4.

O tratamento endovascular é o método de tratamento preferencial, exceto em caso de suspeita de isquemia visceral, pacientes com a anatomia vascular hostil, ou quando houve falha de tratamento endovascular prévio. Nesses casos, é indicada a abordagem aberta^2^^,^ 3.

Relatamos um tratamento endovascular de um volumoso AAPDi verdadeiro, sem estenoses significativas (estenose > 60%) nos troncos arteriais principais, utilizando navegação superseletiva com microcateteres e embolização com micromolas.

## DESCRIÇÃO DO CASO

Uma mulher de 62 anos, hipertensa, diabética tipo 2, obesa e dislipidêmica, estava em investigação ambulatorial por empachamento pós-prandrial com gastroenterologista. Negava história familiar ou pregressa de doenças reumatológicas, assim como episódios de hemorragia digestiva, dor pós-prandial, traumas abdominais, ingestão de bebidas alcoólicas, história prévia de pancreatite, ou perda de peso no período. A angiotomografia demonstrou volumoso AAPDi, com 54 x 60 mm, conectando a AMS com a artéria gastroduodenal. A AMS não apresentava placa ostial, e o TC apresentava placa ostial determinando estenose aproximada de 20% (Figura 1). Foi realizado ecocardiograma transtorácico sem evidências de alterações patológicas.

**Figura 1:** (A) Reconstrução angiotomográfica volumétrica em oblíqua anterior esquerda demonstrando volumoso aneurisma de artéria pancreatoduodenal inferior; (B) Reconstrução volumétrica angiotomográfica em oblíqua anterior direita demonstrando comunicações com tronco celíaco (TC), via artéria gastroduodenal, e com artéria mesentérica superior, via artéria pancreatoduodenal inferior; (C) Corte angiotomográfico axial demonstrando placa ostial de TC; (D) Corte angiotomográfico sagital demonstrando placa ostial em TC determinando estenose aproximada de 20%.

Uma arteriografia seguida de navegação superseletiva com microcateteres e embolização com micromolas foi a opção terapêutica adotada. O procedimento foi realizado em sala de hemodinâmica, sob anestesia local e sedação consciente, com acesso femoral e uso de introdutor 5 Fr (Terumo Medical, Somerset, EUA). Após estudo arteriográfico com cateterização sequencial e seletiva do TC e da AMS, optou-se por cateterização da AMS e navegação superseletiva com microcateter via artéria pancreatoduodenal inferior, do volumoso aneurisma. Foi realizada embolização com uso do Microcateter Rebar-18® (ev3; Plymouth, EUA) e do microfio-guia SilverSpeed-16® (ev3; Plymouth, Minnesota, EUA), além de embolização com 6 micromolas Axium® Prime 3D (ev3; Plymouth, EUA) destacáveis de 18 mm x 40 cm. Após completa embolização do aneurisma, foi realizado novo estudo arteriográfico seletivo via TC e AMS, que revelou completa exclusão do AAPDi, assim como melhora da perfusão das alças intestinais (Figura 2).

**Figura 2:** (A) Arteriografia via artéria mesentérica superior (AMS), com cateterização seletiva; (B) Seletivação com microcateter do volumoso aneurisma da artéria pancreatoduodenal inferior; (C) Arteriografia seletiva da AMS pós-embolização com micromolas, demonstrando exclusão completa do saco aneurismático; (D) Arteriografia seletiva do tronco celíaco, pós-embolização com micromolas, demonstrando exclusão completa do saco aneurismático.

O pós-operatório foi realizado em unidade de terapia intensiva, com boa evolução, e houve alta hospitalar no segundo dia após o procedimento. O controle por angiotomografia após 1 mês mostrou perviedade da AMS e TC, com exclusão completa do AAPDi. Durante o acompanhamento ambulatorial de 1 ano, a paciente apresentou melhora das queixas pós-prandiais previamente referidas (Figura 3).

**Figura 3:** Reconstrução angiotomográfica volumétrica pós-operatória demonstrando ausência de enchimento do aneurisma, com sua exclusão completa.

## DISCUSSÃO

O AAPDi é extremamente raro, mas provavelmente existe subnotificação, uma vez que seus sintomas são inespecíficos e sua localização dificulta o diagnóstico^5^^,^ 6. Acomete ambos os sexos em igual incidência^5^^,^ 6, e a média de idade de maior frequência diagnóstica é aos 60 anos^6^. A principal causa dos AAPDi verdadeiros é a oclusão ou a estenose de um dos troncos arteriais principais, seja AMS ou TC, sendo este último o mais comumente acometido (80%); além disso, o aneurisma está relacionado com a doença arterial oclusiva dos óstios desses troncos em 50-80% dos casos^3^^,^ 7 ^,^ 8. Após esse evento, a velocidade do fluxo sanguíneo se eleva para as arcadas arteriais pancreáticas, justificando a dilatação progressiva do lúmen arterial (hipótese de circulação colateral hiperdinâmica)^2^^,^ 5 ^,^ 8 ^,^ 9. Outras causas relacionadas são aterosclerose, colagenoses e infecções^5^^,^ 10. Estas últimas foram consideradas por muitos anos como a principal causa de AAPDi (60%); no entanto, estudos recentes revelam que a doença aterosclerótica é a causa mais comum, sendo a etiologia infecciosa responsável por 4,8% dos casos diagnosticados, e não mais da metade, como se acreditava anteriormente^10^. A paciente em questão foi diagnosticada com AAPDi verdadeiro, pois não apresentou critérios para suspeição de infecção micótica ou de colagenoses, e também não apresentava estenose ostial de TC ou AMS, afastando a hipótese de circulação colateral hiperdinâmica^2^^,^ 5 ^,^ 8 ^,^ 9. Diante disso, a provável causa do aneurisma dessa paciente é a doença aterosclerótica.

A principal complicação associada ao AAPDi é a ruptura, cuja incidência e mortalidade variam de 45 a 62% e de 21-26%, respectivamente^3^^,^ 11 ^,^ 12. Uma parcela significativa dos diagnósticos dessa afecção é realizada na emergência, com sintomas de epigastralgia ou dor abdominal que irradia para o dorso, os quais podem estar associados a sinais de choque ou hemoperitônio secundários à rotura aneurismática^10^. Essa complicação independe da dimensão do aneurisma^9^.

A paciente relatada apresentou queixa gastrointestinal de empachamento em investigação ambulatorial, com posterior diagnóstico de AAPDi após angiotomografia de abdome. Dor abdominal inespecífica, náuseas, vômitos, icterícia e sangramento gastrointestinal são sintomas que podem estar associados nesses casos. Aneurismas de origem infecciosa podem ocasionar febre. Ao exame físico, uma massa pulsátil em abdome ou sopro abdominal podem ser encontrados, porém são raros. A frequente presença dessas manifestações em pacientes com AAPDi os distingue daqueles com outros tipos de aneurismas de artérias esplâncnicas, em sua maioria assintomáticos (90%)^10^; entretanto, o diagnóstico na maioria dos casos é realizado só após a rotura do aneurisma. Por isso, diante da sua alta mortalidade, o AAV deve ser um diagnóstico diferencial para todo paciente com dor abdominal súbita associada a anemia^9^. Os exames complementares que auxiliam no diagnóstico são: ultrassonografia-Doppler abdominal, que constitui um método disponível e de baixo custo; angiografia por ressonância magnética; e angiotomografia (exame padrão-ouro), que fornece informações necessárias tanto para a o diagnóstico como para a decisão terapêutica^9^^,^ 10.

Por se tratar de afecção rara, seu tratamento tem indicações controversas na literatura, sendo necessária a avaliação de fatores como idade, apresentação clínica, características do aneurisma, comorbidades associadas e fatores de risco^3^^,^ 5 ^,^ 10. Alguns consensos entre os autores quanto à indicação de correção de aneurismas verdadeiros são: diâmetro superior a 20-25 mm, mesmo assintomático; pacientes sintomáticos; pacientes do sexo feminino em idade fértil; e aneurismas associados a processo inflamatório ou infeccioso, doença do tecido conjuntivo, vasculite ou doenças congênitas, devido ao alto risco de rotura^5^^,^ 6 ^,^ 9 ^,^ 13 ^,^ 14. Já o pseudoaneurisma deve ser abordado de imediato após o seu diagnóstico, devido ao elevado risco de rotura^5^^,^ 7 ^,^ 9. Como opções terapêuticas disponíveis, têm-se o tratamento cirúrgico convencional (reparo aberto) e a abordagem endovascular. A cirurgia convencional é indicada para pacientes hemodinamicamente instáveis, quando há necessidade de revascularização do fluxo sanguíneo, e em casos de aneurisma em territórios inacessíveis pela técnica endovascular ou de falha após embolização. Essa técnica consiste na ligadura proximal e distal da artéria, exérese do aneurisma e reconstrução da artéria. O reparo aberto, além de ser um procedimento mais invasivo, apresenta limitações, devido à dificuldade de acesso ao aneurisma, além de estar associado a taxas de mortalidade entre 15-23,7%^5^^,^ 7 ^,^ 10.

Com a evolução das técnicas e dos dispositivos disponíveis, o tratamento endovascular com a embolização do aneurisma tornou-se o tratamento de escolha nesses casos, devido à sua alta efetividade (67-100%), menor taxa de mortalidade (2,7%), menor risco de rotura, menor dor pós-operatória, menor tempo de recuperação após o procedimento, com consequente retorno mais precoce às atividades cotidianas, além de constituir uma opção para pacientes com histórico de cirurgias abdominais que tenham um abdome hostil^5^^,^ 9 ^,^ 10. No entanto, alguns determinantes limitam o seu uso: baixa disponibilidade em centros de emergência, risco de lesões secundárias ao acesso e embolização de órgãos-alvo, assim como elevado custo^9^.

As opções de embolização envolvem a utilização de cianocrilato, molas e stents revestidos ou convencionais, podendo, em algumas ocasiões, serem associados^7^^,^ 9 ^,^ 10. Outras opções de tratamento percutâneo são o desvio de fluxo e a injeção percutânea de trombina^9^^,^ 15 ^,^ 16. A escolha da técnica baseia-se na necessidade de preservar a artéria nutridora, nas características anatômicas e no tipo do aneurisma, além da tortuosidade da artéria nativa. O tratamento adequado do aneurisma é garantido pela embolização completa de todas as artérias de entrada e saída^9^^,^ 15 ^,^ 16.

A paciente deste relato apresentou um AAPDi oligossintomático com dimensões de 54 × 60 mm, sem rotura. Diante da sua compatibilidade com os critérios de indicação para a abordagem terapêtica mais difundidos na literatura, e também pelo elevado risco de rotura, foi indicado o tratamento eletivo por meio da técnica endovascular, com utilização de 6 micromolas Axium® Prime 3D destacáveis (ev3; Plymouth, Minnesota, USA), com trombose permanente no saco aneurismático e exclusão deste. A embolização foi completa, confirmada no intraoperatório pela angiografia seletiva do TC e da AMS, com melhora do fluxo sanguíneo intestinal e boa evolução pós-operatória, inclusive com melhora dos sintomas clínicos prévios. Angiotomografia de controle com 1 mês foi realizada confirmando a trombose total do aneurisma e sem evidências de enchimento deste.
